# Activation of Astrocytic μ-opioid Receptor Elicits Fast Glutamate Release Through TREK-1-Containing K2P Channel in Hippocampal Astrocytes

**DOI:** 10.3389/fncel.2018.00319

**Published:** 2018-09-27

**Authors:** Dong Ho Woo, Jin Young Bae, Min-Ho Nam, Heeyoung An, Yeon Ha Ju, Joungha Won, Jae Hyouk Choi, Eun Mi Hwang, Kyung-Seok Han, Yong Chul Bae, C. Justin Lee

**Affiliations:** ^1^Center for Neural Science and Center for Functional Connectomics, Korea Institute of Science and Technology (KIST), Seoul, South Korea; ^2^Animal Model Research Center, Korea Institute of Toxicology, Korea Research Institute of Chemical Technology, Daejeon, South Korea; ^3^Department of Anatomy and Neurobiology, School of Dentistry, Kyungpook National University, Daegu, South Korea; ^4^Department of Science in Korean Medicine, Graduate School, Kyung Hee University, Seoul, South Korea; ^5^KU-KIST Graduate School of Converging Science and Technology, Korea University, Seoul, South Korea; ^6^Division of Bio-Medical Science & Technology, Korea Institute of Science and Technology (KIST) School, Korea University of Science and Technology, Seoul, South Korea; ^7^Department of Biological Sciences, Korea Advanced Institute of Science and Technology (KAIST), Daejeon, South Korea

**Keywords:** astrocyte, μ-opioid receptor, glutamate, TREK-1, hippocampus

## Abstract

Recently, μ-opioid receptor (MOR), one of the well-known Gi-protein coupled receptors (Gi-GPCR), was reported to be highly expressed in the hippocampal astrocytes. However, the role of astrocytic MOR has not been investigated. Here we report that activation of astrocytic MOR by [D-Ala^2^,N-MePhe^4^,Gly-ol]-enkephalin (DAMGO), a selective MOR agonist, causes a fast glutamate release using sniffer patch technique. We also found that the DAMGO-induced glutamate release was not observed in the astrocytes from MOR-deficient mice and MOR-short hairpin RNA (shRNA)-expressed astrocytes. In addition, the glutamate release was significantly reduced by gene silencing of the TREK-1-containing two-pore potassium (K2P) channel, which mediates passive conductance in astrocytes. Our findings were consistent with the previous study demonstrating that activation of Gi-GPCR such as cannabinoid receptor CB1 and adenosine receptor A1 causes a glutamate release through TREK-1-containing K2P channel from hippocampal astrocytes. We also demonstrated that MOR and TREK-1 are significantly co-localized in the hippocampal astrocytes. Furthermore, we found that both MOR and TREK-1-containing K2P channels are localized in the same subcellular compartments, soma and processes, of astrocytes. Our study raises a novel possibility that astrocytic MOR may participate in several physiological and pathological actions of opioids, including analgesia and addiction, through astrocytically released glutamate and its signaling pathway.

## Introduction

μ-opioid receptor (MOR), one of the three subtypes of opioid receptors, has been believed to be mainly expressed in the interneurons of various brain regions such as ventral tegmental area, nucleus accumbens, and hippocampus. Due to the fact that MOR is a well-known Gi-linked G-protein coupled receptor (Gi-GPCR), the main action of MOR has been believed to be mediated by disinhibition of dopaminergic or glutamatergic neurons through an inhibition of N- and P/Q-type voltage-gated calcium channels via Gαi-mediated decrease of cyclic adenosine monophosphate (cAMP)-dependent protein kinase activity (Rhim and Miller, 1994) or postsynaptic hyperpolarization via the opening of G-protein coupled inwardly-rectifying potassium (GIRK) channels in the GABAergic interneurons (Torrecilla et al., 2002). Meanwhile, the role of astrocytic MOR has not been investigated due to the lack of knowledge about the presence of astrocytic MOR. However, a very recent report clearly demonstrated that MOR is highly expressed in the hippocampal astrocytes, especially in the soma and processes of the astrocytes (Nam et al., 2018).

Astrocytes have been known to fine-tune the synaptic transmission through glutamate release. The glutamate released from astrocytes has been known to exert an excitatory action through binding to group I metabotropic glutamate receptors (mGluR; Perea and Araque, 2007) or *N*-methyl-D-aspartate (NMDA) receptors (Lee et al., 2007; Han et al., 2013). We previously reported that astrocytic glutamate, a major excitatory transmitter in the brain, is released in two distinct modes, which are fast and slow modes through TREK-1 and Best-1 channels, respectively (Woo et al., 2012). The fast release of glutamate from astrocytes is triggered by activation of Gi-GPCR and subsequent dissociation of Gβγ which binds to TREK-1 to open the channel. Among various kinds of Gi-GPCRs, GABA_B_ receptor, cannabinoid receptor CB1, and adenosine receptor A1 were confirmed to elicit a fast glutamate release through TREK-1 from hippocampal astrocytes (Woo et al., 2012). In the current study, we set out to determine the role of newly identified astrocytic MOR and test whether astrocytic MOR activation causes fast glutamate release.

## Materials and Methods

### Animals

Wild-type (WT) C57BL/6 mice, GFAP-green fluorescent protein (GFP; Stock No. 003257), MOR knockout (KO; Stock No. 007559), and MOR-mCherry (Stock No. 029013) mice were obtained from Jackson Laboratory. For primary culture of hippocampal astrocytes, P0–P3 C57BL/6J mice were used. Experimental groups were balanced in terms of animal age, sex and weight. Animals were genotyped before experiments, and they were all caged together and treated in the same way. Animals were randomly and evenly allocated to each experimental group. This study was carried out in accordance with the recommendations of institutional guidelines, Animal Care and Use Committee of KIST (Seoul, South Korea). The protocol was approved by the Animal Care and Use Committee of KIST. Animals were randomly used for experiments.

### Primary Astrocyte Culture

The cerebral cortex of GFAP-GFP mouse or MOR-KO mouse was dissected free of adherent meninges, minced and dissociated into single cell suspension by trituration through a Pasteur pipette. Dissociated cells were plated onto either 12-mm glass coverslips or six-well plates coated with 0.1 mg/mL poly d-lysine (PDL). Cells were grown in Dulbecco’s modified Eagle’s medium (DMEM; Gibco) supplemented with 25 mM glucose, 10% heat-inactivated horse serum, 10% heat-inactivated fetal bovine serum, 2 mM glutamine and 1,000 units/mL penicillin–streptomycin. After 3 days later, cells were vigorously washed with repeated pipetting using medium and the media was replaced to get rid of debris and other floating cell types.

### Preparation of Acutely Dissociated Hippocampal Astrocytes

To deliver MOR-short hairpin RNA (shRNA) or TREK-1-shRNA into the hippocampal cornu ammonis 1 (CA1), we injected 2 μL of lentivirus carrying pSicoR-MOR-shRNA-katushka or pSicoR-TREK-1-shRNA-mCherry into hippocampal CA1 of WT mice (coordinates, AP = −1.85 mm; ML = ±1.6 mm; DV = −1.6 mm). To obtain astrocytes from shRNA-carrying lentivirus-injected hippocampus, after 7 days from virus injection, C57BL/6 mice were deeply anesthetized with isoflurane and decapitated. The brain was rapidly removed and submerged in an ice-cold oxygenated artificial cerebrospinal fluid (aCSF). The hemisected brain was sliced horizontally at 350 μm thickness using vibrating microtome (Leica VT1000s). The hippocampal CA1 region of each slice was mechanically dissociated with a tip of vibrating polished glass pipette, connected to an alternating electronic relay switch (Omron G2R-2-S) under the control of function generator (EZ digital, South Korea) at 1 KHz sine wave function. Five minutes after mechanical dissociation, the aCSF solution containing dissociated cells were collected and centrifuged at 1,000 rpm for 5 min. Collected cells were plated on 0.1 mg/mL PDL-coated cover glass, and incubated in the astrocyte culture media for about 2 h before use. As shown previously (Nam et al., 2018), the viruses used in this study could infect neurons in addition to astrocytes and could thus down-regulate the expression of MOR which is also expressed in hippocampal interneurons. However, this preparation procedure of acutely dissociated hippocampal cells, which includes a vigorous mechanical stimulation by vibrating glass pipet and incubation in the astrocyte culture media, makes neurons to be difficult to survive, while astrocytes are less vulnerable.

### Sniffer Patch

Astrocytes, which were co-cultured with HEK293T cells (obtained from ATCC) transfected with GluR1-L497Y for 3–8 h, were incubated with 5 μM Fura-2AM (mixed with 5 μL of 20% Pluronic acid; P3000MP, Invitrogen) for 40 min and washed at room temperature and subsequently transferred to a microscope stage for imaging. Co-culture with HEK293T cells did not induce any alteration in astrocytic reactivity (Supplementary Figure S1). HEK293T cells were regularly tested for mycoplasma contamination. External solution contained (in mM): 150 NaCl, 10 HEPES, 3 KCl, 2 CaCl_2_, 2 MgCl_2_, 5.5 glucose, pH adjusted to pH 7.3. Intensity images of 510 nm wavelength were taken at 340 nm and 380 nm excitation wavelengths using either iXon EMCCD (DV887 DCS-BV, ANDOR Technology). Two resulting images were used for ratio calculations in Axon Imaging Workbench version 6.2 (Axon Instruments). GluR1LY-mediated currents were recorded from HEK cells expressing GluR1-L497Y under voltage clamp (Vh = −70 mV) using Multiclamp 700B amplifier (Molecular Devices), acquired with pClamp 9.2. Recording pipettes were filled with (mM): 110 Cs-Gluconate, 30 CsCl, 0.5 CaCl_2_, 10 HEPES, 4 Mg-ATP, 0.3 Na3-GTP and 10 BAPTA (pH adjusted to 7.3 with CsOH). For simultaneous recording, Imaging Workbench was synchronized with pClamp 9.2.

### Immunostaining

For immunohistochemistry, animals were deeply anesthetized using 2% avertin and perfused with 0.1 M phosphate-buffered saline (PBS), followed by 4% paraformaldehyde. Brains were post-fixed in 4% paraformaldehyde at 4°C for 24 h and 30% sucrose 4°C for 48 h. Brains were then cut in coronal sections of 30 μm on a cryosection. The sections were blocked in 0.1 M PBS containing 0.3% Triton X-100 (Sigma) and 2% donkey serum (Genetex) for 30 min at room temperature. Primary antibody used are as follow: chicken anti-GFAP (1:500, ab5541, Millipore), rabbit anti-TREK-1 (1:100, APC-047, Alomone Labs), and rabbit anti-MOR (1:200, sc-15310, Santa Cruz Biotechnology). The brain samples with primary antibodies were incubated overnight at 4°C. Then, the sections were washed three times in 0.1 M PBS and incubated in proper secondary antibodies from the Jackson Laboratory for 1.5 h. After three rinses in 0.1 M PBS and DAPI staining at 1:3,000 (PIERCE), the sections were mounted on polysine microscopic slide glass (Thermo Scientific).

For immunocytochemistry, we fixed the cultured astrocytes with 4% paraformaldehyde at 4°C for 10 min. After washing with 0.1 M PBS three times, we performed immunocytochemistry according to the same procedures as immunohistochemistry.

### Confocal Microscopy and Super Resolution Microscopy

For structured illuminated microscopy (SIM), prepared samples were imaged on the Nikon N-SIM-S system which is combined to Nikon Ti2-E microscope with 100× magnification, 1.49 numerical aperture (NA), 1.515 refractive index TIRF objective lens. The PSF width was 0.129 μm. We used TI2-FL N-SIM 405/488/561/640 Quad Band DM filter cube. Images were captured by 2048 × 2048 pixels using Hamamatsu ORCA-Flash 4.0 digital CMOS camera and reconstructed by using Nikon NIS Element version 5.01. SIM images were taken by 93 steps from 10.96 μm total thickness.

For confocal microscopy, images were acquired using a Nikon A1R confocal microscope with 60× magnification lens (CFI Plan Apochromat VC 60× Oil, NA 1.40). The PSF width was 1.00 μm and the pinhole was set to 1.0 airy unit.

### Image Quantification

For co-localization analysis of SIM image, we utilized the colocalization panel in NIS Element version 5.01 and colocalization module of Imaris 9.2. 2D histogram threshold was automatically calculated by built-in algorithm in Imaris 9.2 Colocalization module, which is developed by Costes and Lockett at the National Institute of Health, HCI/SAIC (Costes et al., 2004). To display the 2D histogram, we utilized the co-localization panel in NIS Element HC version 5.01. Automatically calculated threshold intensities of MOR-mCherry and TREK-1 signals from Imaris 9.2 were applied to this histogram. Pearson’s coefficient was automatically calculated within in co-localized volume using Imaris 9.2.

For co-localization analysis of confocal images, we utilized colocalization panel in NIS Element version 5.01. The region of interest was created using auto-detect ROI tool from the maximal intensity projection GFAP images. Pearson’s coefficient in each ROI was automatically calculated from each z-plane image (from total three images stacked) by NIS Element version 5.01 program and averaged.

To quantitate the intensity of GFAP immunoreactivity, we randomly made a fixed set of 10 rectangle-shaped ROIs using rectangular selection tool in ImageJ. Then we measured the GFAP intensity in each ROI.

### Electron Microscopic Immunohistochemistry

For immunostaining for GFP/MOR, TWIK-1/MOR or TWIK-1/TREK-1, three GFAP-GFP transgenic mice (20–25 g) were used. Animals were deeply anesthetized with sodium pentobarbital (80 mg/kg, i.p.) and perfused transcardially with 10 mL heparinized normal saline, followed by 50 mL freshly prepared mixture of 4% paraformaldehyde and 0.01% glutaraldehyde in 0.1 M phosphate buffer (PB), pH 7.4. Hippocampus was removed and postfixed in the same fixative for 2 h at 4°C. The sections were cut sagittally on a Vibratome at 60 μm and cryoprotected in 30% sucrose in PB overnight at 4°C. The sections were frozen on dry ice for 20 min, thawed in PBS (0.01 M, pH 7.4) to enhance penetration. They were pretreated with 1% sodium borohydride for 30 min to quench glutaraldehyde and then blocked with 3% H_2_O_2_ for 10 min to suppress endogenous peroxidases and with 10% normal donkey serum (NDS, Jackson ImmunoResearch, West Grove, PA, USA) for 30 min to mask secondary antibody binding sites. For double immunostaining for GFP and MOR, TWIK-1 and MOR, or TWIK-1 and TREK-1, sections of hippocampus pretreated as above were incubated overnight in a mixture of antibodies. Primary antibodies used are as follow: mouse anti-GFP (1:400, MAB3580, Millipore, Temecula, CA, USA), rabbit anti-MOR (1:2,500, RA10104, Neuromics, Edina, MN, USA), goat anti-TWIK-1 (1:100, sc-11483, Santa Cruz Biotechnology), and rabbit anti-TREK-1 (1:100, APC-047, Alomone Labs Ltd., Jerusalem, Israel) antibodies. After rinsing in PBS, sections were incubated with a mixture of biotinylated donkey anti-mouse (1:200, Jackson ImmunoResearch, West Grove, PA, USA) and 1 nm gold-conjugated donkey anti-rabbit (1:50, EMS, Hatfield, PA, USA) or a mixture of biotinylated donkey anti-goat (1:200, Jackson ImmunoResearch, West Grove, PA, USA) and 1 nm gold-conjugated donkey anti-rabbit (1:50, EMS) antibodies for 2–3 h. The sections were postfixed with 1% glutaraldehyde in PB for 10 min, rinsed in PB several times, incubated for 4 min with HQ silver enhancement solution (Nanoprobes, Yaphank, NY, USA), and rinsed in 0.1 M sodium acetate and PB. The sections were incubated with ExtrAvidin peroxidase (1:5000, Sigma, St. Louis, MO, USA) for 1 h and the immunoperoxidase was visualized by nickel-intensified 3,3’-diaminobenzidine tetrahydrochloride (DAB). The sections were further rinsed in PB, osmicated (in 0.5% osmium tetroxide in PB) for 1 h, dehydrated in graded alcohols, flat-embedded in Durcupan ACM (Fluka, Buchs, Switzerland) between strips of Aclar plastic film (EMS), and cured for 48 h at 60°C. Chips containing prominent staining for MOR, TREK-1 or TWIK-1 in the hippocampus were cut out of the wafers and glued onto blank resin blocks with cyanoacrylate. Serially cut thin sections were collected on Formvar-coated single-slot nickel grids and stained with uranyl acetate and lead citrate. Grids were examined on a Hitachi H7500 electron microscope (Hitachi, Tokyo, Japan) at 80 kV accelerating voltage. Images were captured with Digital Micrograph software driving a cooled CCD camera (SC1000; Gatan, Pleasanton, CA, USA) attached to the microscope, and saved as TIFF files. For the quantitative analysis on the MOR expression in different compartment of astrocyte, 80 electron micrographs (at 25,000 original magnification) were taken in each section of the hippocampal CA1 from each of three mice. Frequency of MOR^+^ soma, process and microdomain of their total numbers within the total areas in each mice were calculated. For the quantitative analysis on the MOR or TREK-1 expression in different compartment of astrocyte showing TWIK-1 immunoreactivity, 80 electron micrographs (at 25,000 original magnification) were taken in each section of the hippocampal CA1 from each of three mice. Gold particle density (number of gold particles/μm^2^) for MOR^+^/TWIK-1^+^ or TREK-1^+^/TWIK-1^+^ soma, process and microdomain in each mouse were calculated.

### Statistical Analysis

Statistical analyses were performed using Prism 7 (GraphPad Software, Inc., San Diego, CA, USA). Differences between two different groups were analyzed with the two-tailed Student’s unpaired *t*-test. For assessment of change of a group by a certain intervention, the significance of data was assessed by the two-tailed Student’s paired *t*-test. For comparison of multiple groups, one-way analysis of variance (ANOVA) with Tukey’s or Dunnett’s multiple comparison test was assessed. Data from multiple independent experiments was assumed to be normal variance. *P* < 0.05 was considered to indicate statistical significance throughout the study. The significance level is represented as asterisks (*P < 0.05, **P < 0.01, ***P < 0.001; ns, not significant). All data are presented as mean ± SEM. No statistical method was used to predetermine sample size. Sample sizes were determined empirically based on our previous experiences or the review of similar experiments in literatures. All experiments were done with at least three biological replicates.

## Results

### MOR Activation Induces Fast Glutamate Release From Hippocampal Astrocytes

We previously reported that astrocytic glutamate, which modulates neuronal excitability and synaptic plasticity (Halassa et al., 2007; Lee et al., 2007; Perea and Araque, 2007), can be released through TREK-1-containing two-pore potassium (K2P) channels, by direct binding of Gβγ which is dissociated from various Gi-GPCRs, in a Ca^2+^-independent manner (Woo et al., 2012). Because MOR is one of the classical Gi-GPCRs, we hypothesized that astrocytic MOR activation would lead to a release of glutamate from astrocytes.

**Figure 1:**
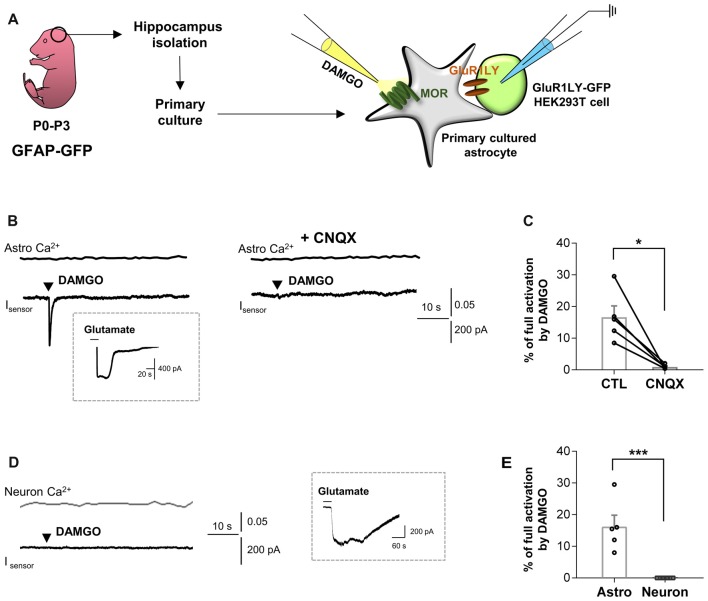
μ-opioid receptor (MOR) activation elicits fast glutamate current from hippocampal astrocytes. **(A)** Schematic diagram for sniffer-patch with primary cultured hippocampal astrocytes prepared from GFAP-green fluorescent protein (GFP) mouse and GluR1LY-GFP-expressing HEK293T biosensor cells. **(B)** Ca^2+^ response (Astro Ca^2+^) induced by brief (0.1 ms) pressure application of 10 μM [D-Ala^2^,N-MePhe^4^,Gly-ol]-enkephalin (DAMGO) from a pipette in acutely dissociated hippocampal astrocyte (upper trace) and simultaneously recorded inward current (I_sensor_) from GluR1-L497Y expressing HEK293T cells (lower trace). DAMGO-induced inward current was inhibited by cyanquixaline (CNQX; 10 μM) application. Inset figure indicates an example trace of full activation current by bath application of 1 mM glutamate to estimate the total surface expression of GluR1-L497Y. **(C)** Summary bar graph for DAMGO-induced glutamate current normalized by the full activation current. The data were expressed as means ± SEM. Data from two independent mice. Paired *t*-test (*P < 0.05). **(D)** Ca^2+^ response (Neuron Ca^2+^) induced by brief (0.1 ms) pressure application of 10 μM DAMGO from a pipette in acutely dissociated hippocampal astrocyte (upper trace) and simultaneously recorded inward current (I_sensor_) from GluR1-L497Y expressing HEK293T cells (lower trace). Inset figure indicates an example trace of full activation current by bath application of 1 mM glutamate to estimate the total surface expression of GluR1-L497Y. **(E)** Summary bar graph for DAMGO-induced glutamate current normalized by the full activation current Unpaired *t*-test (***P < 0.001). The data of astrocytes is identical to the control group in **(C)**. The data were expressed as means ± SEM. Data from two independent mice. Paired *t*-test (***P < 0.05).

To test this hypothesis, we first performed the sniffer-patch using primary cultured hippocampal astrocytes co-cultured with biosensor HEK293T cells. The HEK293T cells were transfected with a non-desensitizing form of α-amino-3-hydroxy-5-methyl-4-isoxazolepropionic acid (AMPA) receptor subunit (GluR1-L497Y) for detecting glutamate release from astrocytes, as previously described (Lee et al., 2007; Woo et al., 2012). The cells were stained with Fura-2AM (5 μM in 5 μL of 20% pluronic acid) for Ca^2+^ imaging. To activate astrocytic MOR, we puffed [D-Ala^2^,N-MePhe^4^,Gly-ol]-enkephalin (DAMGO), a selective MOR agonist, for 0.1 ms by using a glass pipette placed close to the astrocyte (Figure 1A). We found that DAMGO treatment causes a fast glutamate release from astrocytes, which was detected as an inward current from the sensor cell with no detectable change in intracellular Ca^2+^ concentration in astrocytes (Figure 1B). These findings indicate that the MOR activation induces glutamate release in a Ca^2+^-independent manner. To test if the DAMGO-induced current is indeed mediated by the sensor, we utilized a selective AMPA receptor blocker, cyanquixaline (CNQX; 10 μM). We found that the DAMGO-induced inward current was completely blocked by application of CNQX, indicating that the DAMGO-induced current is indeed the sensor-mediated current (Figures 1B,C; ctl, 16.64 ± 3.548; CNQX, 0.9288 ± 0.3022). To estimate the concentration of released glutamate, we applied 1 mM glutamate to fully activate GluR1-L497Y channels to obtain the percentage of full activation (Figure 1B, inset), which can be used to convert the normalized sensor current (16.64%) to a glutamate concentration of 1.76 μM based on the known concentration-dependence of GluR1-L497Y for glutamate, as previously described (Figure 1C; Lee et al., 2007; Woo et al., 2012). The glutamate current was significantly and completely blocked by CNQX treatment (Figure 1C), suggesting that the DAMGO-induced inward current is indeed elicited by glutamate binding. On the other hand, this DAMGO-induced glutamate current was not observed from primary cultured hippocampal neurons (Figures 1D,E; astro, 16.64 ± 3.548; neuron, 0.000 ± 0.000).

**Figure 2:**
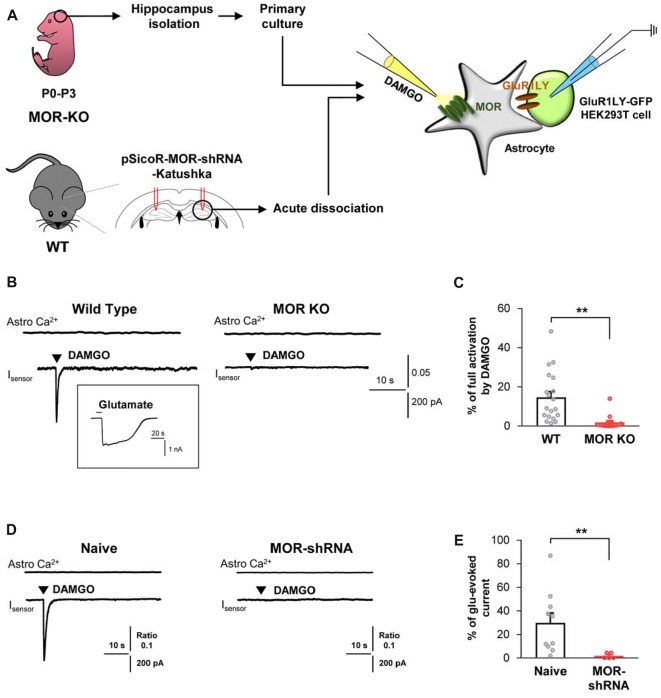
DAMGO-induced astrocytic glutamate release is caused by MOR activation. **(A)** Schematic diagram for sniffer-patch with primary cultured hippocampal astrocytes prepared from MOR knockout (KO) mouse or acutely dissociated astrocytes from pSicoR-MOR-shRNA-Katushka-injected hippocampus of wild-type (WT) mouse. **(B)** Representative traces of Ca^2+^ response and inward current induced by DAMGO in primary cultured hippocampal astrocyte of WT and MOR KO mice. Inset indicates an example trace of full activation current induced by bath application of 1 mM glutamate. **(C)** Summary bar graph for DAMGO-induced glutamate current normalized by the full activation current in WT and MOR KO mice. Numbers in the bar graph indicate the number of cells tested from at least three independent mice for each group. Unpaired *t*-test (**P < 0.01). **(D)** Representative traces of Ca^2+^ response and inward current naïve or MOR-shRNA-infected astrocyte. **(E)** Summary bar graph for DAMGO-induced glutamate current normalized by the full activation current in naive and MOR-shRNA-infected astrocytes. Numbers of tested cells are indicated on each bar. Unpaired *t*-test with Welch’s correction (**P < 0.01).

Next, to test if the DAMGO-induced glutamate release is also observed in the astrocytes from adult brains, we obtained acutely dissociated CA1 hippocampal cells from the acute brain slices of an adult mice, consisting of mainly astrocytes (Figure 2A). We found that DAMGO-induced MOR activation also caused fast glutamate release from the acutely dissociated astrocytes, detected as an inward current whose kinetics are analogous to those from cultured astrocytes. The glutamate release was not accompanied by changes in intracellular Ca^2+^ concentration in astrocytes (Figure 2B). In MOR-KO mice, we could not find any significant DAMGO-induced glutamate current from the hippocampal astrocytes from adult brains (Figures 2B,C; WT, 14.66 ± 2.945; KO, 1.852 ± 0.9922). Next, we performed gene-silencing of MOR using lentivirus carrying pSicoR-MORshRNA-katushka, which was previously developed and validated by immunohistochemistry and western blotting methods (Supplementary Figure S2A; Nam et al., 2018). Gene-silencing of MOR also significantly reduced the DAMGO-induced inward current (Figures 2D,E; naïve, 29.87 ± 8.42; MOR-shRNA, 1.904 ± 1.036). Meanwhile, the full-activated inward currents by glutamate (1 mM) application were not significantly different in all groups (Supplementary Figure S3). These findings indicate that DAMGO-induced glutamate release is indeed MOR-dependent.

### Glutamate Release Upon MOR Activation Is Mediated by TREK-1 Channel

**Figure 3:**
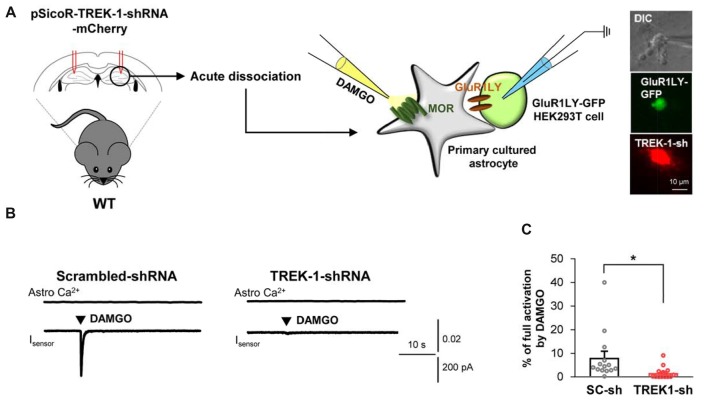
Astrocytic glutamate release upon MOR activation is mediated by TREK-1. **(A)** Left, schematic diagram of sniffer-patch with acutely dissociated astrocytes from pSicoR-TREK-1-shRNA-mCherry-injected hippocampus and GluR1LY-GFP-expressing HEK cells. Right, differential interference contrast image and fluorescent image of GluR1LY-GFP-expressing HEK cell (green) and TREK-1-shRNA-mCherry-infected hippocampal astrocyte (red). **(B)** Representative traces of Ca^2+^ response and inward current in acutely dissociated hippocampal astrocyte of scrambled-shRNA and TREK-1-shRNA-injected mouse. **(C)** Summary bar graph for DAMGO-induced glutamate current normalized by the full activation current in scrambled shRNA and TREK-1-shRNA-injected mice. Data from at least three independent mice for each group. Unpaired two-tailed *t*-test with Welch’s correction (*P < 0.05).

Based on the previous report demonstrating that glutamate release caused by the activation of astrocytic Gi-GPCRs, such as CB1 and A1, is mediated by TREK-1-containing K2P channels (Woo et al., 2012), we tested if MOR-dependent astrocytic glutamate release is also mediated by TREK-1-containing K2P channels. We performed the sniffer-patch technique from acutely dissociated hippocampal astrocytes that were infected with lentivirus carrying TREK-1-shRNA or scrambled-shRNA, which were previously validated *in vitro* (Woo et al., 2012) and *in vivo* (Supplementary Figure S2B). We found that DAMGO-induced glutamate release from the TREK-1-shRNA-infected astrocytes was significantly less than that from the scrambled-shRNA-infected astrocytes (Figure 3; scrambled, 8.077 ± 2.784; TREK-1-shRNA, 1.658 ± 0.6445). Taken together, these findings suggest that MOR activation causes fast glutamate release through a TREK-1-containing K2P channel from hippocampal astrocytes.

### MOR and TREK-1 Are Co-localized in Soma and Processes of Hippocampal Astrocytes

**Figure 4:**
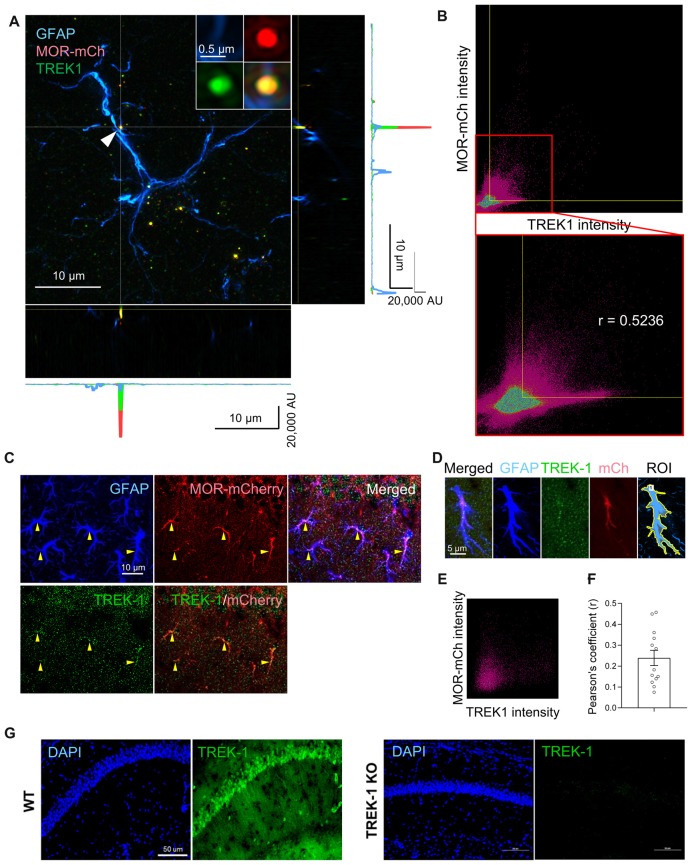
MOR and TREK-1 are co-expressed in hippocampal astrocytes of MOR-mCherry mouse. **(A)** A structured illumination microscopic (SIM) image of a hippocampal astrocyte of MOR-mCherry mouse, immunostained with antibodies against GFAP and TREK-1. **(B)** 2D histogram of mCherry intensity and TREK-1 intensity in the SIM image **(A)**. Pearson’s coefficient (r) is calculated within the colocalized pixels which were automatically thresholded by Imaris 9.2 program. **(C)** Representative confocal images of co-localization of MOR-mCherry and TREK-1 in astrocytes in hippocampal cornu ammonis 1 (CA1) of MOR-mCherry mice. **(D)** An example of ROI generation within a single astrocyte. **(E)** Representative 2D histogram of mCherry intensity and TREK-1 intensity in the confocal image. **(F)** Pearson’s coefficients (r) between mCherry and TREK-1 from confocal images of single astrocytes.** (G)** A confocal image of hippocampal tissues from WT and TREK-1 KO mice, stained with an antibody against TREK-1.

To utilize the TREK-1-containing K2P channels as the molecular machinery of glutamate release upon MOR activation, MOR and TREK-1 proteins should be close to each other in the same compartment of an astrocyte. To test this hypothesis, we performed immunohistochemistry with anti-TREK-1 antibody using MOR-mCherry mice, in which the reporter mCherry was tagged to the C-terminus of MOR (Erbs et al., 2015). To precisely analyze the co-localization of MOR and TREK-1, we performed SIM with these tissues. We could find a significant population of MOR-mCherry-positive spots and TREK-1-positive spots in or near the GFAP-positive signals in the SIM image (Figure 4A). Next, to quantitatively estimate the co-localization between MOR and TREK-1, we made a two dimensional histogram of MOR-mCherry and TREK-1 signal intensities in each pixel. After automatic thresholding by Imaris 9.2, we calculated the Pearson’s coefficient in co-localized pixels, which was 0.5236 (Figure 4B), indicating a moderate co-localization between MOR and TREK-1. In addition, to confirm their co-localization in more hippocampal astrocytes in a simpler way, we performed confocal microscopy with the TREK-1-stained hippocampal tissues from MOR-mCherry (Figure 4C). Consistently, we observed a moderate co-localization of MOR and TREK-1 from 13 additional astrocytes with an average Pearson’s coefficient of 0.2393 (Figures 4D–F). On the other hand, the Pearson’s coefficient between GFAP-positive and NeuN-positive signals in a CA1 hippocampal tissue, which are known to be well separated with each other, was 0.000 (data not shown). The specificity of TREK-1 antibody was validated by the observation that TREK-1-positive signals were absent in TREK-1 KO mice (Figure 4G). These findings indicate that MOR-mCherry and TREK-1 are significantly co-localized in hippocampal astrocytes.

**Figure 5:**
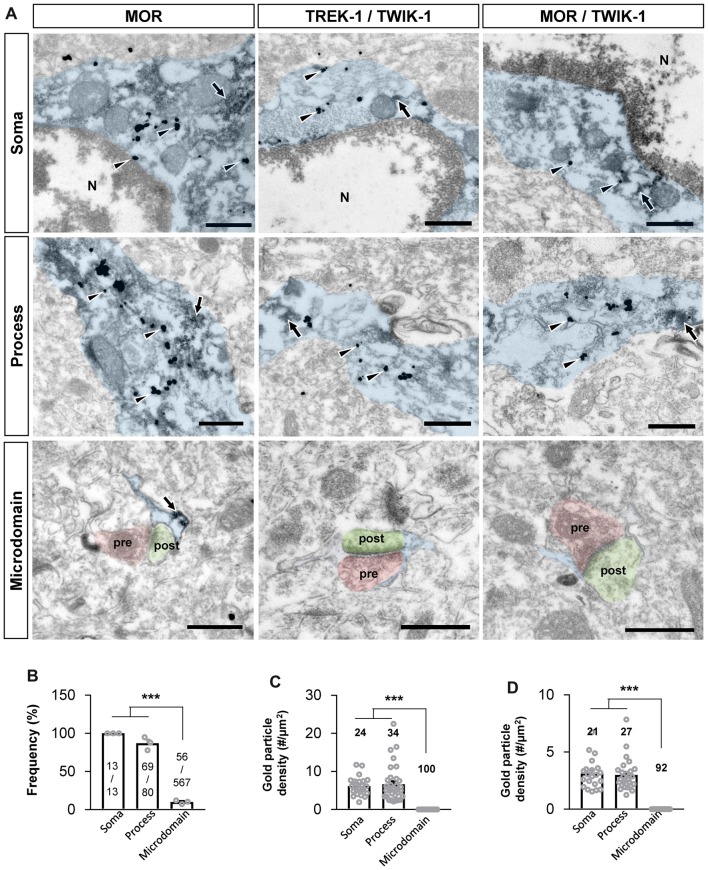
MOR and TREK-1 co-localizes in astrocytic soma and process. **(A)** Subcellular distribution (soma, process and microdomain) of MOR in astrocyte (left). MOR is stained with immunogold with silver enhancement (dark specks, arrowheads), and GFP, representing astrocyte, is stained with immunoperoxidase (dark amorphous deposits, arrows). Cellular distribution of double labeling of TREK-1/TWIK-1 (middle) and MOR/TWIK-1 (right) in astrocyte. TREK-1 or MOR is stained with immunogold with silver enhancement (dark specks, arrowheads), and TWIK-1 is stained with immunoperoxidase (dark amorphous deposits, arrows). The soma, process, and microdomain of the astrocyte was colored blue. Presynaptic axon terminal (pre) and postsynaptic dendrite (post) were colored red and green, respectively. N is nucleus. Scale bar indicates 500 nm. **(B)** Summary bar graph for the frequency of MOR detection in each subcellular structure of astrocytes. Numbers in the bar graph are the number of MOR-positive soma, processes or microdomains out of total number of profiles observed. The data was collected from three animals. One-way analysis of variance (ANOVA) with Tukey’s multiple comparison test (***P < 0.001). **(C,D)** Summary bar graph for the gold particle density of TREK-1 **(C)** or MOR **(D)** in each subcellular structure of TWIK-1-positive astrocytes. Numbers in the bar graph indicate the number of images analyzed from three different animals. One-way ANOVA with Tukey’s multiple comparison test (***P < 0.001).

Next, we sought to confirm if MOR and TREK-1-containing K2P channel are co-localized in same subcellular compartments, because MOR and K2P channel should be physically close to each other for the coupling of MOR activation and glutamate release through K2P channel. Recently, MOR in the hippocampal astrocytes has been reported to be localized in the soma and processes by an electron microscopy (EM) study (Nam et al., 2018). We first re-confirmed the subcellular localization of MOR in astrocytes by performing EM immunogold labeling using an anti-MOR antibody (targeting the C-terminus of MOR, 1359–1403), whose specificity to MOR has been previously validated with MOR-deficient mice (Nam et al., 2018). We also found that MOR was highly expressed in the soma and processes of astrocytes rather than in the perisynaptic microdomains, as indicated by the frequency of appearance (Figures 5A,B). To investigate the ultrastructural colocalization of MOR and TREK-1 in astrocytic substructures *in vivo*, we performed sequential labeling of immunoperoxidase and immunogold. Due to an overlap of hosts for the two primary antibodies, we found it difficult to directly label both MOR and TREK-1. Instead, based on our previous study demonstrating that TREK-1-containing K2P channels mediate the passive conductance channels in astrocytes by forming a heterodimer with TWIK-1 subunit (TREK-1-TWIK-1 heterodimer; Hwang et al., 2014), we double-stained TREK-1 with TWIK-1 and, separately, MOR with TWIK-1, by utilizing TWIK-1 immunoperoxidase-labeling as a common denominator. EM images showed that both TREK-1-TWIK-1 (Figures 5A,C) and MOR/TWIK-1 (Figures 5A,D) were co-labeled in the soma and processes of astrocytes rather than in the microdomains. These findings suggest that MOR and TREK-1- and TWIK-1-containing K2P channels localized in the same subcellular compartment and that MOR activation induces glutamate release through nearby K2P channels.

## Discussion

In the current study, we demonstrated that astrocytic MOR activation causes TREK-1-mediated fast glutamate release from hippocampal astrocytes. We utilized sniffer patch technique to directly detect the glutamate released upon MOR activation from both primary cultured hippocampal astrocytes and acutely dissociated hippocampal astrocytes. We also confirmed that the glutamate-induced current was not observed in either MOR-shRNA condition or MOR-KO mice. We further demonstrated that MOR and TREK-1-containing K2P channels are co-localized in the soma and processes in the hippocampal astrocytes by performing immunohistochemistry and EM.

How can MOR-mediated astrocytic glutamate participate in the synaptic transmission? In a previous report, we demonstrated that astrocytic glutamate released through TREK-1 channel targets both NMDA receptors and mGluRs in neighboring neurons when the distance between neuronal membrane and astrocytic TREK-1 is 10–40 nm (Woo et al., 2012). Therefore, the location of TREK-1 is critical for the mode of astrocytic glutamate’s action. In the current study, we found that TREK-1-containing K2P channels are not localized in the perisynaptic microdomains, but localized in the soma and processes. Based on the previous reports that mGluRs, but not NMDA receptors, are localized in the presynaptic axonal processes, our findings raise a possibility that astrocytic glutamate might target neuronal mGluRs to modulate synaptic transmission.

We also demonstrated that MOR-induced glutamate release was Ca^2+^-independent. Nonetheless, our study does not exclude the possibility that MOR (Gi-GPCR)-induced astrocytic glutamate could be released via a Ca^2+^-dependent pathway, as previously shown with CB1 and GABA_B_ (Nilsson et al., 1993; Kang et al., 1998; Navarrete and Araque, 2008). It is also possible that CB1- and GABA_B_-induced glutamate release could share the same pathway that was presented in this study. Future studies are needed to clarify the role of activation of other astrocytic Gi-GPCR, such as D2, β2-adrenergic receptor, group II mGluRs, and 5-HT_1_, in modulating neural circuit and synaptic transmission.

Even though we demonstrated that activation of astrocytic MOR causes glutamate release to modulate synaptic activity, a fundamental curiosity about the physiological and pathological roles of astrocytic MOR in the hippocampus still remains. Recently, accumulating lines of evidence have suggested that hippocampus might play a critical role in the development of addiction-related behaviors (McQuiston and Saggau, 2003; Tzschentke and Schmidt, 2003; Black et al., 2004; Kelley, 2004; Atkins et al., 2008; Roy et al., 2017). For example, optogenetic inhibition of axon terminals of dorsal subiculum (dSub) neurons projecting to medial entorhinal cortex layer 5 was shown to impair the retrieval of cocaine-associated contextual memory formed in the conditioned place preference paradigm (Roy et al., 2017). Moreover, glutamate, in addition to dopamine, has been implicated in the opioid addiction through participating in formation of opioid-associated memory (Popik and Wróbel, 2002; Veeneman et al., 2011; Peters and De Vries, 2012). Therefore, astrocytic MOR might play a critical role in the formation of opioid-associated memory through glutamate release in the CA1 hippocampus. These new ideas await future investigations.

## Author Contributions

CL, YB and K-SH designed the study. DW and YJ conducted sniffer patch experiments. JB and YB conducted electron microscopy (EM). JW conducted structured illumination microscopy. M-HN, HA, JC, and EH conducted immunohistochemistry. DW, CL and M-HN wrote the manuscript. All authors contributed to analysis and discussion of the results.

## Conflict of Interest Statement

The authors declare that the research was conducted in the absence of any commercial or financial relationships that could be construed as a potential conflict of interest.

## Abbreviations

aCSF: artificial cerebrospinal fluid
AMPA: a-amino-3-hydroxy-5-methyl-4-isoxazolepropionic acid
CA1: cornu ammonis 1
cAMP: cyclic adenosine monophosphate
CNQX: cyanquixaline
DAB: 3,3’-diaminobenzidine tetrahydrochloride
DAMGO: [D-Ala^2^,N-MePhe^4^,Gly-ol]-enkephalin
GFP: Green Fluorescent Protein
Gi-GPCR: Gi-protein coupled receptors
GIRK: G-protein coupled inwardly-rectifying potassium
K2P: two-pore potassium
mGluR: metabotropic glutamate receptors
MOR: μ-opioid receptor
NMDA: N-methyl-D-aspartate
shRNA: Short hairpin RNA
PB: phosphate buffer
PBS: phosphate-buffered saline.
