# Supplementary material for: Activation of Astrocytic μ-opioid Receptor Elicits Fast Glutamate Release Through TREK-1-Containing K2P Channel in Hippocampal Astrocytes
Source: Front Cell Neurosci. 2018 Sep 27;12:319. doi: 10.3389/fncel.2018.00319 (PMC6170663; doi:10.3389/fncel.2018.00319)
Supplement: Supplementary file 1 [file Data_Sheet_1.docx]

**Figure S1. Astrocytic reactivity was not altered by co-culture with GluR1LY-GFP-transfected HEK293T cells.** Box-and-whisker graph shows 5 to 95 percentiles. The numbers on the graph indicate the numbers of ROIs analyzed. Significance was analyzed by Student’s t-test.


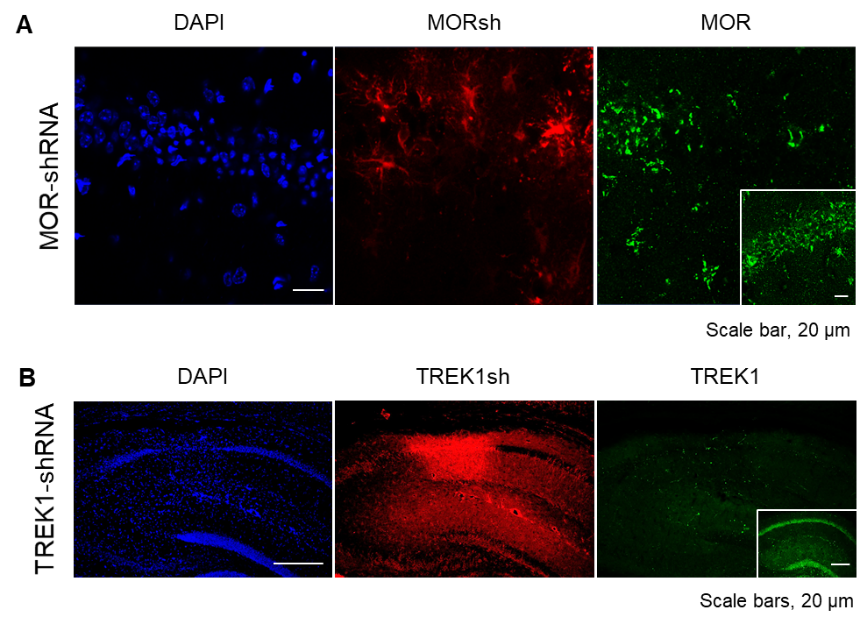


**Figure S2. *In vivo* knockdown efficiency of MOR-shRNA and TREK1-shRNA. (A)** Lentivirus carrying MOR-shRNA markedly reduced the hippocampal MOR expression *in vivo*, compared to scrambed-shRNA (inset). **(B)** Lentivirus carrying TREK1-shRNA markedly reduced the hippocampal TREK1 expression *in vivo, compared to* scrambed-shRNA (inset).

**Figure S3. Glutamate (1 mM)-induced maximal inward currents over the all experimental groups.** 1C, 2C, 2E, and 3C indicate each figure number. Significance was analyzed by One-way ANOVA with Tukey’s multiple comparison test.
